# Understanding the Biology of Human Interstitial Cells of Cajal in Gastrointestinal Motility

**DOI:** 10.3390/ijms21124540

**Published:** 2020-06-25

**Authors:** Daphne Foong, Jerry Zhou, Ali Zarrouk, Vincent Ho, Michael D. O’Connor

**Affiliations:** 1 School of Medicine, Western Sydney University, Campbelltown, NSW 2560, Australia; 19243317@student.westernsydney.edu.au (D.F.); j.zhou@westernsydney.edu.au (J.Z.); v.ho@westernsydney.edu.au (V.H.); 2 Campbelltown Private Hospital, Campbelltown, NSW 2560, Australia; surgery@southwestsurgery.com.au

**Keywords:** gastrointestinal motility, peristalsis, interstitial cells of Cajal, ICC, molecular characterisation, bioinformatics, mouse, human, pluripotent stem cells.

## Abstract

Millions of patients worldwide suffer from gastrointestinal (GI) motility disorders such as gastroparesis. These disorders typically include debilitating symptoms, such as chronic nausea and vomiting. As no cures are currently available, clinical care is limited to symptom management, while the underlying causes of impaired GI motility remain unaddressed. The efficient movement of contents through the GI tract is facilitated by peristalsis. These rhythmic slow waves of GI muscle contraction are mediated by several cell types, including smooth muscle cells, enteric neurons, telocytes, and specialised gut pacemaker cells called interstitial cells of Cajal (ICC). As ICC dysfunction or loss has been implicated in several GI motility disorders, ICC represent a potentially valuable therapeutic target. Due to their availability, murine ICC have been extensively studied at the molecular level using both normal and diseased GI tissue. In contrast, relatively little is known about the biology of human ICC or their involvement in GI disease pathogenesis. Here, we demonstrate human gastric tissue as a source of primary human cells with ICC phenotype. Further characterisation of these cells will provide new insights into human GI biology, with the potential for developing novel therapies to address the fundamental causes of GI dysmotility.

## 1. Introduction

Gastrointestinal (GI) motility disorders can occur throughout the length of the gut—from the oesophagus to the colon. Accordingly, GI motility disorders can present with a range of chronic symptoms, including nausea and vomiting, that greatly affect a patient’s quality of life [1]. Difficulties in obtaining large amounts of human GI tissue for research have limited our understanding of GI motility. Currently, there are no cures for GI motility disorders, and symptom management primarily relies on lifestyle changes and medications.

The GI tract is an essential system for the digestion of food, absorption of nutrients and release of waste. These processes occur in different segments of the GI tract, requiring the movement of content between segments. This movement is facilitated by active and passive peristalsis—coordinated slow waves of muscle contraction and relaxation [2,3]. Cells responsible for GI motility involve smooth muscle cells, enteric neurons, and interstitial cells, including telocytes and interstitial cells of Cajal (ICC). A range of studies suggest that ICC act as specialised GI pacemaker cells [4,5]. For example, abnormalities in ICC numbers and structure have been associated with several GI motility disorders such as gastroparesis [6,7]. While ICC are recognised as essential for normal GI motility, the fundamental molecular biology of human ICC in normal or diseased GI function is yet to be fully defined. As a consequence, the development of novel therapies for GI motility disorders has been restricted.

In this review, we discuss the differences known between human and mouse ICC and present data demonstrating enrichment of human ICC-like cells from primary gastric tissue. We also examine gaps in the current understanding of human ICC—their sources, molecular characterisation, and potential involvement in GI disorders and therapy—in order to propose a framework for better understanding the biology of human ICC in GI motility.

## 2. Anatomical Locations of ICC

Originally described as ‘primitive neurons’ by Santiago Ramón y Cajal [8], ICC have since been shown to be mesenchymal-derived cells. ICC are primarily located within the muscle layer of the GI tract, and while they are somewhat morphologically heterogeneous [9], they are typically characterised by elongated cell bodies with several cell processes. Various studies have noted similarities between ICC and smooth muscle cells, as they share common ultrastructural features including caveolae and a basal lamina [10,11,12,13]. An abundance of mitochondria has also been observed within ICC close to the endoplasmic reticulum (ER) and plasma membrane. This led to the suggestion that the mitochondria form part of the pacemaker system within ICC to provide energy for regulating ICC membrane potential [14]. Close associations between adjacent ICC as well as with other GI cells also support the hypothesis that ICC may regulate GI motility. ICC interactions with neighbouring ICC, with smooth muscle cells (via gap junctions), and with enteric neurons (via nerve varicosities) have been described throughout the GI tract within mice and humans [12,15,16,17,18,19,20]. An additional gut cell type—previously called telocytes and now referred to as platelet-derived growth factor receptor alpha-positive (PDGFRα^+^) cells—has also been observed in close proximity to ICC (reviewed in [21,22]). These PDGFRα^+^ cells are morphologically similar to ICC and were initially referred to as ‘ICC-like cells’. Although PDGFRα^+^ cells are considered to be distinct from ICC, the relationship between ICC and PDGFRα^+^ cells, as well as their role in GI motility, are yet to be fully investigated.

### ICC Subtypes

In the 1990s, a cell surface receptor tyrosine kinase—KIT (also known as KIT proto-oncogene, c-KIT and CD117)—was identified as an ICC marker in the GI tract [23]. This discovery was instrumental in enabling detailed study of ICC throughout the GI tract [24,25,26,27,28]. It has since been shown that KIT signalling (via stem cell factor; SCF) is necessary for the development and maintenance of mature functional ICC, and that ICC are required for the initiation of electrical activity in the GI tract [29,30,31]. However, KIT is not exclusively expressed on ICC. Other, non-ICC cell types, such as macrophages and mast cells, also express high levels of KIT [25,27,32]. This has led to a search for additional ICC markers, and more recently, anoctamin 1 (ANO1)—a calcium-activated chloride channel—has been validated as a more selective ICC marker in the GI tract [32]. Moreover, ANO1 has been shown to be required for generating slow-wave activity in GI muscles [33,34].

ICC can also be categorised into subtypes based on their varying locations, morphology, functions, and cell-specific markers within the GI tract—with more molecular details understood for mouse ICC, as shown in Table 1 and reviewed by Komuro et al. [9,35]. For example:

- Myenteric ICC (ICC-MY) are located surrounding the circumference of the myenteric plexus. They are multipolar cells with branched processes that form a network around the myenteric plexus. ICC-MY have been suggested to be the primary pacemaker cells of the stomach and small intestine muscles, involved in generating and propagating slow-wave activity through smooth muscle cells [4].
- Intramuscular ICC (ICC-IM) are found within the circular and longitudinal muscle. They are bipolar or spindle-shaped cells that align with the long axis of surrounding smooth muscle cells. ICC-IM play a key role in mediating enteric neurotransmission [36].
- ICC of the deep myenteric plexus (ICC-DMP) are found exclusively in the small intestine. They are multipolar cells that are closely associated with nerve bundles of the DMP. Similar to ICC-IM, ICC-DMP also mediate enteric neurotransmission [36]. Neurokinin-1 receptor (NK1R) has been used as a marker to identify and isolate murine Kit^+^ ICC-DMP [28].
- Other ICC subtypes have been described, including submucosal ICC (ICC-SM; located at the interface between the submucosa and circular muscle of the stomach), subserosal ICC (ICC-SS; found between the serosa and longitudinal muscle of small intestine and colon) and septal ICC (ICC-SEP; located between and surrounding muscle bundles, particularly in larger animals like humans). ICC-SEP have also been implicated in propagating pacemaker activity into muscle bundles of the human jejunum [45]. More recently, a small stem cell-like population of ‘ICC progenitors’ were defined as ‘Kit^low^CD44^+^CD34^+^Insr^+^Igf1r^+^’ within the mouse stomach [38].

## 3. Potential Sources of ICC

Together with their dispersed anatomical locations, the relatively small numbers of ICC within the gut has made investigating ICC biology highly challenging. As a result, a number of ICC sources have been investigated, each with its own advantages and disadvantages.

### 3.1. Murine GI Tissue

Murine GI tissues have been widely utilised as both in vitro and in vivo sources of ICC. Early research observed Kit^+^ cells within primary GI cultures, although these studies were limited as the cells began to lose Kit immunoreactivity and tended to trans-differentiate into a smooth muscle cell-like phenotype [25,26]. Nonetheless, this approach has been used to demonstrate that immuno-labelling with anti-Kit and anti-Ano1 antibodies have consistently identified ICC throughout gastric (stomach), small intestine and colon tissue [25,27,28,32,46]. Furthermore, several mouse models have been established to study ICC under normal and abnormal motility conditions. Ro et al. [39] were the first group to develop a transgenic model of GFP-labelled ICC (*Kit^+/copGFP^*). These mice have been extensively used to investigate the molecular and functional phenotypes of healthy ICC in the gut. Loss-of-function mutations within the *Kit* or *Scf* locus (e.g., W/W^v^, Sl/Sl^d^ and W(jic)/W(jic)) have been used to study the absence of certain types of ICC throughout the GI muscle, showing phenotypes including disrupted pacemaker activity [23,47,48]. Mice with complete or conditional *Ano1*-knockouts have shown loss of Ano1 immunoreactivity but continued development of Kit^+^ ICC networks, albeit with reduced slow-wave activity [33,49,50]. Mouse models of ICC in GI diseases, including models of diabetic gastroparesis [51] and surgical manipulations [52,53], have also been explored.

### 3.2. Human GI Tissue

Access to sufficient amounts of fresh normal and diseased human GI tissue for research has been a major challenge. Such tissue is often restricted to small amounts obtained from patient biopsies. In some cases, larger quantities of human GI tissue can be accessed from sleeve gastrectomy resections or GI cancer surgeries. Various studies have histologically identified KIT^+^ and ANO1^+^ ICC within gastric tissue from sleeve gastrectomy [32,54,55] and gastric cancer patients [56,57,58]. Similar studies have also been performed on small intestinal tissue from gastric bypass patients [28,32,33,59,60] and colon tissue from colon cancer patients [32,54,61,62]. Despite ICC appearing to display normal morphologies in these GI tissues, the sources of these tissues are not from healthy donors but are representative of a specific population (e.g., obesity or cancer). Moreover, possible variations in clinical characteristics among different patients have been noted. For example, Gomez-Pinilla et al. [54] reported that reduced numbers and collective volumes of KIT^+^ ICC were associated with ageing in both gastric and colon tissue. In other studies, similar ICC numbers were observed between obese and non-obese individuals [63], but increased *KIT* and *ANO1* expression were found in gastric muscle biopsies of obese subjects [64].

### 3.3. Bioengineered GI Tissue

While ICC from primary GI tissues can be harvested and cultured in vitro, loss of cell/tissue phenotype and function typically occurs [25,26,34,41]. Given the potential advantages of maintaining ICC and gut tissue in vitro for functional assessment and drug development, a number of attempts have been made at bioengineering GI tissue. For example, a serum-free method to culture ‘intestinal muscularis complexes’ using cells isolated from primary murine and human intestinal smooth muscle has been described [65]. These primary cultures were maintained for up to 28 days and exhibited a range of gut-like properties. This included spontaneous contractions and the presence of gut cell types including Kit^+^ cells. To further maintain the functionality of ICC in vitro, STO (SIM, Sandos Inbred Mouse) embryonic fibroblast feeder cells have been co-cultured with enriched murine Kit^+^ ICC (purified from primary mouse intestinal smooth muscle cell mixture through the use of magnetic-activated cell sorting and an anti-Kit antibody) [66]. Intriguingly, this model maintained contractility and contained Kit^+^Ano1^+^ cells for up to 14 days. Moreover, these observations were replicated when enriched Kit^+^ ICC were replaced with the primary intestinal smooth muscle cell mixture (containing Kit^+^Ano1^+^ cells). While these bioengineered GI tissues represent an opportunity to harvest large quantities of ICC, they are complex in vitro systems involving several cell types, and the degree to which they mimic primary GI organs in vivo needs to be characterised in more detail.

More recently, advancements in pluripotent stem cell technology have led to the generation of three-dimensional gut organoid units using a multi-stage differentiation protocol that attempts to recapitulate elements of embryonic GI development in vitro. The resulting human gut organoids are reported to be comprised of most gut cell layers, possess functions reminiscent of peristalsis, and display the presence of KIT^+^ cells [67,68]. The potential of this pluripotent stem-cell-based approach, while yet to be fully validated, has been extensively reviewed elsewhere [69,70].

## 4. ICC and Molecular Characteristics

Access to large numbers of ICC is essential for advancing our molecular understanding of ICC biology. ICC characterisation studies have typically relied upon antibody-based purification methods, namely magnetic-activated cell sorting (MACS) and fluorescence-activated cell sorting (FACS) (reviewed in [70]). To date, FACS has proven to be the most effective approach for purifying Kit^+^ ICC from mouse GI tissue [24,27,28,37,39,40] or human GI tissue [55,61]. These isolated ICC have been consistently validated through real-time polymerase chain reaction (RT-PCR) analysis of genes considered to be ICC markers (e.g., *Kit* and *Ano1*).

### 4.1. Murine ICC

The isolation of sufficient numbers of purified murine ICC has led to fairly extensive characterisation via transcriptomics and functional studies (Table 1). Whole transcriptomic expression profiling—such as microarray and RNA-sequencing (RNA-seq)—is a powerful tool for investigating the molecular mechanisms that underpin cellular functions and biological processes. Using microarray-based transcriptomics of FACS-purified small intestinal ICC-MY and ICC-DMP, Chen et al. [24] were the first to show that *Ano1* was a highly expressed gene in ICC. The differential gene expression profiles this study revealed supported the idea of different functional properties of ICC-MY and ICC-DMP, i.e., as pacemakers and mediators of neurotransmission, respectively. More recently, RNA-seq performed on populations of FACS-purified GFP^+^ ICC from the small intestine and colon of *Kit^+/copGFP^* mice demonstrated a potential new set of ICC-specific genes [40]. For example, *Thbs4* and *Hcn4* were identified as two new putative ICC markers. This same group compared their ICC datasets with datasets from smooth muscle cells and Pdgfrα^+^ cells to generate a proposed ‘smooth muscle transcriptome browser’ [71]. However, given that each transcriptional profile arose from pooled cell populations obtained from 30–40 mice, the level of intra-sample heterogeneity of each gut cell type is not known.

Numerous in vivo mouse studies have established a functional role for ICC as GI pacemakers and in GI neurotransmission as reviewed by several authors [4,5,72]. Others have investigated the pacemaker activity of isolated ICC, using electrophysiology and calcium imaging. Early electrophysiology studies demonstrated spontaneous rhythmic activity in Kit^+^ cells within small intestinal cultures, showing the activity of these cells to be similar to intact muscle [41,42]. When MACS-purified Kit^+^ cells from primary small intestinal cultures were re-cultured, electrical pacemaker activity was retained [25]. However, it has been noted that some pacemaker mechanisms may have been lost within primary ICC cultures (e.g., [34,41]). Issues relating to loss of ICC phenotype upon culture can, to some extent, be circumvented by analysis of freshly isolated ICC. Goto et al. [43] identified large inward currents by activating depolarisation in single Kit^+^ cells isolated from the small intestine, although identification of characteristic ICC morphology amongst the Kit^+^ population (that potentially included hematopoietic cells) was challenging. More recent studies made use of GFP^+^ ICC isolated from the small intestine of *Kit^+/copGFP^* mice to more easily identify definitive ICC. Large inward currents were also observed in these cells, and Ano1 was suggested to regulate their pacemaker activity [34]. Furthermore, blocking ryanodine receptors (Ca^2+^ channels on the ER membrane) inhibited slow-wave activity [44]. Collectively, these functional studies on isolated ICC provide evidence that ICC pacemaker activity may be generated by intracellular Ca^2+^ release from the ER within ICC, thereby activating Ano1 channels.

### 4.2. Human ICC

In contrast to our understanding of mouse ICC, the fundamentals of human ICC biology are only emerging from recent studies (Table 2). This, in part, is attributed to the limited access to normal human GI tissue and clinical variability from surgically resected tissue. To date, only one transcriptional analysis of human ICC has been reported: a microarray analysis of gastric-derived, FACS-purified KIT^+^ human ICC [55]. Another study has reported morphological and histological analysis of FACS-purified KIT^+^ ICC progenitors from human colonic cancer resections [61]. Both studies noted the small number of isolated ICC—approximately 1000–10,000 cells per patient sample.

The limited, human-specific detail we have of ICC function has ramifications for the development of new therapies for GI motility disorders. Electrophysiology and calcium imaging have shown similar pacemaker activity in ICC-MY within human small intestinal muscle strips compared to the mouse small intestine [59]. However, other aspects of human ICC biology do not appear to occur in mouse ICC. For example, immunohistochemical identification of freshly isolated mouse ICC has been difficult as the dissociation procedure affected the ability to visually identify ICC [43]. On the other hand, ICC were easier to identify in freshly dissociated human jejunum, whereby ICC processes were preserved [60]. *KIT* mRNA was also detected in individual cells visually identified as ICC. Additionally, the sodium channel, *SCN5A*, mRNA and a Na^+^ current were identified in these ICC that had electrophysiological, pharmacological, and mechano-sensitive properties similar to human jejunum circular smooth muscle. This Na^+^ current was essentially absent in mouse ICC, suggesting that SCN5A activity may represent human-specific ICC biology. In a different study, fluorescent substance P (a ligand for NK1R) was used to histologically identify ICC-DMP in murine and human small intestinal tissue [28]. While substance P fluorescence was selectively found in Kit^+^ ICC-DMP of murine small intestine, a similar attempt to label KIT^+^ ICC-DMP within human small intestine was found to be incomplete and non-exclusive as ICC-IM may have also been labelled. Taken together, these studies indicate a need to further develop and optimise molecular techniques specific for the investigation of human ICC biology.

## 5. ICC and GI Disorders

By defining the biology of ICC within the normal GI tract, some important links have been reported between ICC and GI disorders. Decreased or injured ICC have been documented in several human GI motility disorders, such as ageing [54], diabetic gastroparesis [63,73], Hirschsprung’s disease (HSCR) [74,75], and slow transit constipation [62,76,77]. However, direct translation between animal models and human GI physiology is yet to be fully established. A major limitation has been monitoring ICC loss or dysfunctions in the live human GI tract during disease development and progression. While animal models may be poor predictors of how novel therapies will perform in humans, they nevertheless have been useful in developing cellular and molecular frameworks to use in understanding normal biology and disease pathogenesis in humans.

### 5.1. ICC and Ageing

Alterations in GI motility have been linked to ageing, with a number of GI motility complications (such as constipation) occurring more frequently in the elderly population (reviewed in [78,79,80]). In one of the first studies to investigate the role of ICC in ageing, Gomez-Pinilla et al. [54] sampled gastric and colon tissue from patients aged 25–70 and 36–92 years old, respectively. The number and collective volume of KIT^+^ ICC networks declined by 13% per decade of life in the muscle layer of gastric and colon tissue. Such findings have also been demonstrated in ageing mice. In the stomach, jejunum and colon, Kit^+^ ICC numbers and networks decreased over a 24-month time period, which was correlated with reduced Kit protein levels [81]. Interestingly, loss of ICC was first observed in the stomach, followed by the intestine and colon, indicating that gastric ICC functionality may be the first affected during ageing. Additionally, similar changes in ICC network volumes have been found in the terminal bowel of ageing mice [82]. Whilst thorough molecular information regarding ICC and ageing is yet to be elucidated, these data indicate that a change in ICC functionality may, in part, affect GI motility with age.

### 5.2. ICC and Diabetic Gastroparesis

Reduced KIT^+^ ICC numbers have been reported in the gastric muscles of diabetic patients [56], which is often linked to GI motility disorders such as gastroparesis. In humans, diabetic gastroparesis presents as delayed gastric emptying in the absence of obstruction or any other identifiable, non-diabetic cause [83]. Investigation of type 1 or type 2 diabetic patients has shown approximately that 42% exhibit normal gastric emptying and 58% abnormal emptying (36% with delayed emptying and 22% with accelerated emptying) [84]. Similar outcomes were seen in type 2 diabetic patients with poor glycaemic control; i.e., ~66% had abnormal (though not always symptomatic) gastric emptying [85]. Other studies of gastroparesis have shown ultrastructural changes in ICC and disrupted slow-wave activity within the gastric antrum and corpus, termed ‘ICC-opathy’ (reviewed in [86]). More recently, some studies have suggested a link between inflammation and ICC loss in diabetic gastroparesis patients. Low numbers of CD206^+^ macrophages were found to be correlated to loss of KIT^+^ ICC within the gastric corpus of diabetic gastroparesis patients [87]. Moreover, transcriptomic and proteomic analyses on gastric muscle biopsies from diabetic gastroparesis patients have further supported this hypothesis that innate immune signalling and inflammation may play a role in disease pathogenesis [88,89].

Delayed gastric emptying has also been observed in mouse diabetic models. Interestingly, reduced numbers of gastric Kit^+^ cells and disrupted GI pacemaker activity have been reported in type 2 diabetic (*db/db*) mice [90], as well as diabetic GFP^+^ ICC (*Kit^+/copGFP^; Lep^ob/ob^*) mice [39] and non-obese diabetic gastroparesis (NOD) mice [51]. Furthermore, a loss of Ho-1 (a cytoprotective molecule against oxidative stress) up-regulation was associated with decreased gastric Kit protein expression and delayed gastric emptying in NOD mice, suggesting a role of oxidative stress in diabetes [91]. Interestingly, administration of interleukin-10 (which activates the expression of Ho-1 protein) into NOD mice led to increased gastric emptying and more organised gastric Kit^+^ ICC networks [92]. In a different study, depleted ICC in NOD mice were also accompanied by reduced Scf production and smooth muscle atrophy [93]. Moreover, in organotypic gastric muscle cultures, such ICC loss was inhibited by insulin or Igf-1 via rescue of smooth muscle cells and *Scf* expression, suggesting a potential role of smooth muscle cells in diabetic-associated loss of ICC. On the other hand, in female obese type 2 diabetic leptin receptor-mutant (*Lepr*^db/db^) mice, hyperglycaemia and hyperinsulinemia were observed with elevated oxidative stress, rapid gastric emptying and increased gastric Kit^+^ ICC [94]. Thus, while diabetes may be associated with both ICC gain and loss, the mechanisms underpinning these phenomena in humans are yet to be fully explored.

### 5.3. ICC and Other GI Motility Disorders

ICC loss has also been observed along with disrupted slow-wave activity in mouse models of intestinal surgical resection [52] and small bowel obstruction [53]. Interestingly, after removing the insult, ICC networks and slow-wave activity began to recover.

Hirschsprung’s disease (HSCR) is a congenital colon disorder that causes chronic constipation. Altered KIT^+^ ICC networks have been documented in the bowel [75] and colon [74] of HSCR patients. Additionally, reduced ANO1 immunoreactivity and protein levels were found in the colon of HSCR patients [95]. Intriguingly, in human colon samples from both normal and HSCR patients, Kit^low^CD34^+^Igf1r^+^ candidate ‘ICC progenitors’ were identified at a frequency of 0.7% of total cells [61] (similar to the Kit^low^CD34^+^Igf1r^+^ candidate ‘ICC progenitors’ identified in the mouse stomach [38]). Reduced proportions of mature and progenitors of ICC were reported, along with ultrastructural ICC injury, in the narrow part of the HSCR colon.

### 5.4. ICC and Gastrointestinal Stromal Tumours (GISTs)

Gastrointestinal Stromal Tumours (GISTs) are the most common mesenchymal tumour of the gut. Some 60–70% of GISTs occur in the stomach and contain activating mutations in the *KIT* or *PDGFRA* genes [96]. GISTs have been treated with imatinib, a tyrosine kinase inhibitor, but many patients eventually develop imatinib-resistance.

One hypothesis for the development of GISTs is that they may arise from KIT^+^CD34^+^ ICC in the human GI tract [97,98]. However, this hypothesis is controversial as others have reported that KIT^+^ ICC are distinct from CD34^+^ cells [99,100]. Nonetheless, in mouse models of GIST, knock-in mutations in the *Kit* gene (K641E and KIT-Asp818Tyr) resulted in hyperplastic ICC and the development of GISTs, suggesting ICC may be involved in disease pathogenesis [101,102]. Lőrincz et al. [38] isolated Kit^low^CD44^+^CD34^+^Insr^+^Igf1r^+^ ‘ICC progenitors’ (representing 0.6% of the total cells) by FACS from mouse gastric tissue. Interestingly, these cells were able to expand with Scf and differentiate into mature functional ICC with Igf-1 in vitro. Transplantation of this cell population into nude mice showed that these ‘ICC progenitors’ induced the formation of malignant tumours containing Kit^+^Ano1^+^ cells [103]. Furthermore, in vitro assessment of these cells indicated they were resistant to imatinib as well as being resistant to neutralising antibodies against Kit and Scf—treatments that would normally inhibit proliferation of mature ICC. A 2016 human microarray study showed that genes in the hedgehog signalling pathway, molecular regulators of GI mesenchymal development, were altered within GIST tissue compared to FACS-purified gastric-derived ICC [55]. It was suggested that dysregulated hedgehog signalling—possibly within ICC—may be involved in GIST development. At present, these data suggest a potential role of ICC in GIST pathogenesis. In particular, the mouse data suggests that ‘ICC progenitors’ may be GIST precursors and potential targets for GIST therapy.

## 6. ICC and GI Therapy

It is clear from numerous studies that loss of normal ICC functioning is implicated in a range of GI-related diseases. Despite this, the specific role of ICC in disease pathology is still poorly understood—indicating more detailed molecular characterisation of normal and diseased ICC at different stages of disease is required. Such increased knowledge of ICC biology could identify novel drug targets and candidate small molecules to restore impaired ICC function. Towards this end, some initial steps have been made towards regeneration of rudimentary ICC networks and restoration of pacemaker functions, particularly with murine ICC.

### 6.1. Cell-based ICC Therapy

Mouse stem-cell-like ‘ICC progenitors’—isolated from gastric tissue by FACS—have been shown to regenerate and maintain mature ICC networks under certain conditions in vitro [38]. However, as such a cell population is rare, further research is required to identify and characterise these cells in the human GI tract. Nevertheless, these ‘ICC progenitors’, and other possible stem-cell-derived ICC [69,70], may represent a promising route to investigate potential regenerative therapies as well as personalised medicine for GI disorders.

Several groups have investigated ICC transplantation as an alternative approach. One of the first attempts involved allotransplantation of isolated ICC from *Kit^+/copGFP^* mice into small intestinal muscle strips of W/W^v^ mutant mice [104]. This resulted in development of Kit^+^ ICC-MY networks along with functional pacemaker activity. Other groups have used both a different potential source of ICC progenitors and a different method of transplantation. Bone-marrow-derived mesenchymal stem cells have been shown to recolonise injured organs including the intestinal epithelium [105,106]. Interestingly, transplanted mouse GFP^+^ bone marrow-derived cells were able to migrate into the GI tract, where they appeared to differentiate into Kit^+^ ICC-like cells within the myenteric plexus of the small intestine following intestinal injury [107]. Similar findings were also reported along with improved gastric emptying in W/W^v^ mutant mice [108]. However, McCann et al. [109] found that when GFP^+^Kit^+^ bone-marrow-derived cells (from *Kit^+/copGFP^* mice) were transplanted into the small intestine of W/W^v^ mutant mice, they did not observe these cells differentiate into network-forming ICC-like cells. These contradictory findings indicate more detailed molecular and functional characterisation of the transplanted cells is needed before the potential utility of this approach can be properly assessed.

### 6.2. Scaffold-Based ICC Therapy

As ICC are a mesenchymal-derived cell type, a 2018 study seeded mouse mesenchymal stem cells onto a hydrogel scaffold and then placed it onto the luminal side of gastric explants in vitro [110]. Some of the mesenchymal stem cells appeared to successfully migrate into the gastric tissue, as evidenced by Kit^+^ staining. However, typical ICC networks were not seen to develop. When primary cultured mouse ‘intestinal muscularis complexes’ (containing Kit^+^ cells) were combined with electrospun poly-caprolactone scaffold sheets in vitro, spontaneous contractions were observed [65]. Similar findings occurred when electrospun poly-caprolactone scaffold sheets were seeded with STO feeder cells and primary MACS-purified mouse Kit^+^ ICC [66], thus demonstrating attachment and alignment onto the scaffold sheets in vitro. In addition, when a primary mouse intestinal smooth muscle cell mixture was cultured on STO-seeded electrospun poly-caprolactone scaffold sheets, rhythmic contractions developed, and Kit^+^Ano1^+^ cells were observed after 10 weeks. While this approach may represent a more clinically relevant approach, improved in vitro ICC maintenance conditions for scaffolds may need to be established prior to assessment in animal transplantation studies.

## 7. Establishing a Source of Candidate Human ICC for Molecular Characterisation

**Figure 1:**
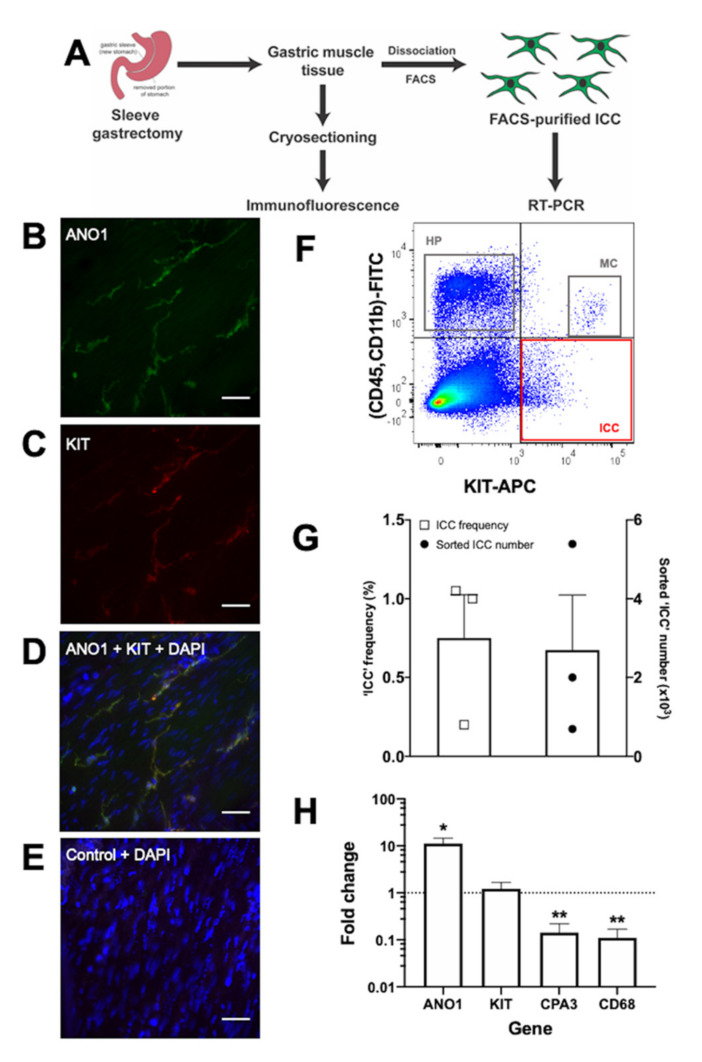
Identification and capture of candidate interstitial cells of Cajal (ICC) from human gastric muscle tissue. (**A**) Outline of the experimental workflow for processing human sleeve gastrectomy samples for immunofluorescence or flow cytometry. (**B**–**E**) Immunofluorescence data obtained from cryosections of human gastric muscle stained with DAPI nuclear stain (blue) and antibodies that detect ICC marker proteins ANO1 (B; green) and KIT (C; red), with overlapping expression observed (D; ANO1+KIT+DAPI). A representative negative control (E; secondary antibody only control+DAPI) shows no background staining, thereby supporting the specificity of the staining patterns seen in B–D. Images were taken at 40× objective on the Zeiss Axio Imager M2 microscope. Scale bar = 50 μm. (**F**) A representative fluorescence-activated cell sorting (FACS) plot illustrating key human sleeve gastrectomy cell populations including: KIT^+^/CD45^-^/CD11b^-^ ICC (red box); hematopoietic cells (HP; KIT^-^/CD45^+^/CD11b^+^) (grey box); and mast cells (MC; KIT^+^/CD45^+^/CD11b^+^) (grey box). (**G**) Plot of ICC frequency and total sorted ICC numbers. Each dot represents an individual patient sample. (**H**) RT-PCR verification of cell marker genes in the FACS-captured ICC population: *ANO1* (ICC), *KIT* (ICC, mast cells), *CPA3* (mast cells), and *CD68* (hematopoietic cells). Data were normalised against GAPDH mRNA levels, expressed relative to unsorted cells (dotted line), and represented as mean fold change ± standard error of the mean from three biological samples. Data were analysed using paired *t*-test; * *p* < 0.05, ** *p* < 0.01.

As a step towards obtaining a large source of human ICC, we have used immunofluorescence and flow cytometry to search for ICC in gastric tissue derived from human sleeve gastrectomy patients (Figure 1A). Immunofluorescence showed KIT^+^/ANO1^+^ cells present within the gastric muscle layer (Figure 1B–E), consistent with previous reports of ICC in human gastric tissue [32,58]. Dissociation of the gastric muscle tissue into single-cell suspensions enabled capture of KIT^+^/CD45^-^/CD11b^-^ cells via flow cytometry (Figure 1F,G), similar to published analysis of gastric-derived FACS-purified human ICC [55]. On average, these candidate ICC represented ~0.8% of the total sorted live cell count, with ~2700 ICC obtained per sample (Figure 1G). Encouragingly, these KIT^+^/CD45^-^/CD11b^-^ candidate ICC were significantly enriched (11-fold) in *ANO1* mRNA expression. They also had retained *KIT* mRNA expression and had reduced (to 0.1-fold) mRNA expression of both the hematopoietic markers *CPA3* and *CD68* (Figure 1H).

**Figure 2:**
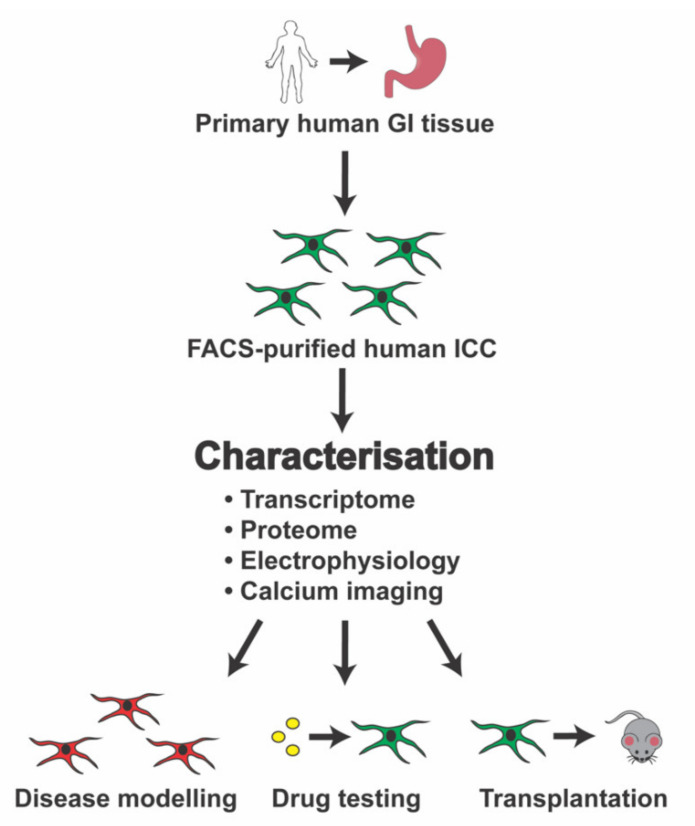
Workflow for characterisation of patient-derived human gastric interstitial cells of Cajal (ICC).

Based on these initial findings, primary human gastric ICC can be obtained in sufficiently high numbers for molecular characterisation via immunofluorescence, transcriptomics, and proteomics, and also functional analyses such as electrophysiology and calcium imaging (Figure 2). Such fundamental characterisation will assist in identifying the transcriptional networks, and also growth factors and signalling pathways, that are important for controlling human ICC biology. In turn, these future studies will help to identify new drug targets (e.g., ion channels) and related candidate drugs relevant to ICC-associated GI disorders. Molecular characterisation of primary human gastric ICC will also provide other useful information, such as identification of transcription factors responsible for maintaining a human ICC subtype. This information could then be used to generate a much larger source of human ICC. For example, through optimisation of human stem-cell-differentiation strategies [67,68,69,70], or through trans-differentiation mediated by over-expression of ICC-expressed transcription factors [111]. A sustainable source of human ICC will help to (i) define the molecular pathology of GI disorders using human cells rather than animal models and (ii) facilitate development of ICC transplantation strategies (with or without cell scaffolds) for GI disorders.

## 8. Conclusions and Future Prospects

Current treatments for GI motility disorders are limited and inadequate. This leads to chronic and debilitating GI symptoms that cause long-term reductions in the quality of life for millions of patients worldwide. While ICC are recognised as necessary for normal GI motility, they also exhibit key pathologies within several GI diseases. Encouragingly, ICC also show potential for regenerative medicine and present an important cellular target for therapy.

Over the last few decades, an extensive body of work has established some fundamental molecular characteristics of murine ICC in both normal and diseased GI tissue. However, it is unclear how closely these findings can be translated to human GI physiology and disease treatment. The scarcity of large sources of normal and diseased human GI tissues for molecular analysis has greatly limited our fundamental understanding of human ICC and their involvement in GI-related diseases. Here, we show that human gastric tissue is an appropriate source of FACS-purified primary human ICC. Future research that focuses on further characterising these primary human ICC will help define mechanistic details of their role in human GI biology. In turn, this will assist in developing potential cell- or drug-based therapies to treat GI disorders.

## Figures and Tables

**Table 1:** Key molecular characterisation studies using interstitial cells of Cajal (ICC) isolated from murine GI tissue.

Ref(s) ^1^	Source	Purification Method	Downstream Technique(s)	Major Finding(s)
[26]	Small intestinal cultures and tissue	Collected Kit^+^ cells through micro-pipette	RT-PCR	Cultured ICC expressed smooth muscle myosin mRNA, while freshly isolated ICC did not
[25]	Small intestinal cultures and tissue	MACS-purified Kit^+^ ICC	Flow cytometry Rhod-2 (calcium) and TMRM imaging	Better purification of Kit^+^ ICC from primary cultures Retained pacemaker activity after re-culturing
[27,37]	Small intestinal and gastric tissue	MACS- and/or FACS-purified Kit^+^ ICC	Flow cytometry RT-PCR	FACS was the most effective method for purifying freshly isolated Kit^+^ ICC depleted of contaminating cells
[28]	Small intestinal tissue	FACS-purified substance P^+^Kit^+^ ICC-DMP	Immunofluorescence RT-PCR	Selectively identified ICC-DMP as fluorescent substance-P-internalising Kit^+^ cells within tissue and cell suspension Purified ICC-DMP expressed *Kit* and *Tacr1* (encodes NK1R)
[24]	Small intestinal tissue	FACS-purified Kit^+^ ICC-MY and substance P^+^ Kit^+^ ICC-DMP	RT-PCR Microarray	First to show *Ano1* as a highly expressed gene in ICC Verified ICC-MY as pacemakers and ICC-DMP as mediators of neurotransmission
[38]	Gastric tissue	FACS-purified Kit^+^ ICC	Flow cytometry	Identified Kit^low^CD44^+^CD34^+^ Insr^+^Igf1r^+^ as ‘ICC progenitors’ Expanded and matured into ICC with SCF and IGF-1 in vitro
[39]	Small intestinal tissue (*Kit^+/copGFP^*)	FACS-purified GFP^+^ cells	RT-PCR	First to develop a GFP-labelled ICC model Verified isolated GFP+ cells as ICC
[40]	Small intestinal and colon tissue (*Kit^+/copGFP^*)	FACS-purified GFP^+^ ICC	RNA-sequencing (RNA-seq)	First ICC RNA-seq datasets Identified potentially novel ICC-specific markers e.g., *Thbs4* and *Hcn4*
[41,42]	Small intestinal cultures	Identified single Kit^+^ ICC	Whole-cell patch clamping	Exhibited rhythmic activity in ICC similar to intact muscle
[43]	Small intestinal tissue	Identified single Kit^+^ ICC	Whole-cell patch clamping	Observed large inward currents in ICC following depolarisation
[34,44]	Small intestinal tissue (*Kit^+/copGFP^*)	Identified single GFP^+^ ICC	Whole-cell patch clamping	Pacemaker activity in ICC may be regulated by intracellular Ca^2+^ via Ano1 channels

^1^ References are representative examples of key literature in the field and thus are not a comprehensive listing.

**Table 2:** Key molecular characterisation studies using interstitial cells of Cajal (ICC) isolated from human GI tissue.

Ref(s) ^1^	Source	Purification Method	Downstream Technique(s)	Major Finding(s)
[60]	Small intestinal tissue (gastric bypass)	Identified single KIT^+^ ICC	Single-cell RT-PCR Whole-cell patch clamping	Individual isolated ICC expressed *KIT* and *SCN5A* Identified Na^+^ current within ICC
[59]	Small intestinal tissue (gastric bypass)	Identified ICC-MY within muscle strips ^2^	Electrophysiology Calcium imaging	Observed pacemaker mechanisms similar to murine ICC
[28]	Small intestinal tissue (gastric bypass)	Identified Substance P^+^ KIT^+^ ICC-DMP	Immunofluorescence	Substance P staining in KIT^+^ ICC-DMP was incomplete and non-exclusive Some ICC-IM may have also been labelled
[55]	Gastric tissue (sleeve gastrectomy)	FACS-purified KIT^+^ ICC	Microarray	Validated isolated ICC by gene expression profiling
[61]	Colon tissue (colon cancer)	FACS-purified KIT^+^ ICC	Flow cytometry Immunofluorescence	Identified Kit^low^CD34^+^Igf1r^+^ and Kit^+^CD34^+^Igf1r^+^ ‘ICC progenitors’ within human colon

^1^ References are representative examples of key literature in the field and thus are not a comprehensive listing. ^2^ Note that experiments were conducted on myenteric interstitial cells of Cajal (ICC-MY) within intact tissue.

## Abbreviations

GI	Gastrointestinal
ICC	Interstitial cells of Cajal
ER	Endoplasmic reticulum
PDGRFα^+^	Platelet-derived growth factor receptor alpha-positive
ICC-MY	Myenteric ICC
ICC-IM	Intramuscular ICC
ICC-DMP	ICC of the deep myenteric plexus
ICC-SM	Submucosal ICC
ICC-SS	Subserosal ICC
ICC-SEP	Septal ICC
MACS	Magnetic-activated cell sorting
FACS	Fluorescence-activated cell sorting
RT-PCR	Real-time polymerase chain reaction
RNA-seq	RNA-sequencing
HSCR	Hirschsprung’s disease
GIST	Gastrointestinal stromal tumour
